# First report of *Providencia rettgeri, Colpodella* spp.*, Ehrlichia* spp.*, and Rickettsia hoogstraalii* in ticks infesting goats of Pakistan

**DOI:** 10.1371/journal.pntd.0014060

**Published:** 2026-03-09

**Authors:** Shakir Ullah, Hafsa Sher, Raquel Cossío-Bayúgar, Ioannis A. Giantsis, Sumbal Haleem, Sadaf Niaz, Michael E. von Fricken, Adil Khan

**Affiliations:** 1 Department of Zoology, Abdul Wali Khan University Mardan, Mardan, Pakistan; 2 Centro Nacional de Investigación Disciplinaria en Salud Animal e Inocuidad, INIFAP, Carretera Federal Cuernavaca‑ Cuautla, Col. Progreso, Jiutepec, Morelos, Mexico; 3 Department of Animal Science, Aristotle University of Thessaloniki, Thessaloniki, Greece; 4 Department of Zoology, Shaheed Benazir Bhutto Women University Peshawar, Peshawar, Pakistan; 5 Department of Environmental & Global Health, University of Florida, Gainesville, Florida, United States of America; 6 Department of Zoology, Bacha Khan University Charsadda, Charsadda, Pakistan; State Key Laboratory of Pathogen and Biosecurity, CHINA

## Abstract

Ticks are the second most important vector of infectious diseases, after mosquitoes, and can transmit several diseases of concern for both human and veterinary health. This study molecularly barcoded ticks collected from goats in Pakistan and screened for associated pathogens. From July 2023 to June 2024, examination of 253 goats (*Capra hircus*) in the 7th district of Khyber Pakhtunkhwa found 170 goats infested with 1,305 ticks, equating to a mean abundance of 5.15 ticks per goat. A phenol-chloroform technique was used to extract DNA and subsequently amplify the presence of pathogen DNA targeting 16S, 18S gltA, and ompA genes. Tick DNA was also amplified for the molecular confirmation of species using 12S rDNA partial sequence. All collected ticks were identified morphologically and molecularly as *Haemaphysalis Punctata* (519), *Hyalomma anatolicum* (380)*, Hae. sulcata* (269), and *Hy. excavatum* (137), including 361 females, 323 males, 286 larvae and 198 nymphs. This study detected several tick-borne pathogens including *Colpodella* spp., *Ehrlichia* spp. and *Rickettsia hoogstraalii*, as well as detecting the bacteria *Providencia rettgeri. Rickettsia hoogstraalii* was found in Haemaphysalis *punctata* collected from Karak District. In contrast, *Hy. excavatum* from Banuu district were found to carry *P. rettgeri*. *Hyalomma excavatum* infesting goats in Buner, Chitral, and *Hy. anatolicum* form Kohistan, District tested positive only for *Colpodella* spp*.* whereas a single species of uncultured *Ehrlichia* spp. was found in Hae. *sulcata* collected from Mansehra, and Lakki Marawat district. This research’s novel report of human pathogenic microbes detected in ticks has implications for livestock and human health, as well as the role ticks potentially play in zoonotic disease transmission in Pakistan.

## Author summary

Ticks are well known for spreading diseases to humans and animals, yet the full range of pathogens they carry in many parts of the world—including Pakistan—remains poorly understood. In this study, we examined ticks collected from goats across seven districts of Khyber Pakhtunkhwa to better understand which species are present and what microbes they may transmit. Using both traditional identification and DNA sequencing, we found four common tick species and discovered several microorganisms of potential concern. These included *Rickettsia hoogstraalii*, *Ehrlichia* species, and *Colpodella* species—an emerging protozoan increasingly linked to human and animal infections. We also detected *Providencia rettgeri*, a bacterium typically associated with hospital acquired infections, marking the first time it has been reported in ticks. Although more research is needed to determine whether ticks can transmit these microbes to humans or livestock, our findings highlight the importance of continued surveillance in regions where people and animals live in close contact. This study provides new insight into the diversity of pathogens circulating in Pakistan’s tick populations and underscores the need for public health awareness and further investigation into their potential risks.

## Introduction

Ticks are considered the most important hematophagous ectoparasite that feed incidentally on humans and are second only to mosquitoes as vectors of human diseases [1,2,3]. Out of the roughly 900 tick species that have been identified, ~ 700 are classified as hard ticks (Ixodidae), ~ 200 as soft ticks (Argasidae), and a single species described as a member of the Nuttalliellidae family [4], many of which carry and transmit tick-borne pathogens (TBPs).

Ticks from the *Hyalomma* genus are significant vectors of viruses, bacteria, and parasites, and are known to transmit Nairovirus, the causative agent of Crimean-Congo hemorrhagic fever in humans [5,6], Wad Medani virus, and Thogoto virus to name a few. Bacterial pathogens vectored by *Hyalomma* ticks include *Ehrlichia*, *Anaplasma*, *Rickettsia*, and *Coxiella*. They also play a key role in the transmission of parasitic pathogens such as *Babesia ovis*, [7,8,9,10]. *Hyalomma excavatum* [11] (Acari: Ixodidae)] parasitizes various domestic animals including dogs, sheep, goats, cattle, camels, and horses [11,12]. In addition to bacterial infections like rickettsiosis, *Hy. excavatum* also spreads protozoan diseases including babesiosis and theileriosis [13,14,15].

Viral infections such as Crimean Congo hemorrhagic fever have received more attention given the severity of disease and observed seasonal spikes coinciding with Eid al-Adha in Pakistan [16,17]. Moreover, anaplasmosis and 60 cases of *Babisia* were found in a nomadic goat herding community [18]

*Rickettsia*, an obligate intracellular Gram-negative bacterium, are primarily transmitted to vertebrates by hard ticks (Ixodidae) and include species with known human pathogenicity and others with unknown pathogenicity [19,20,21,22]. Multiple tick species have been shown to carry *Rickettsia hoogstraalii* in different parts of the world [Brown et al., 2016,^19^,^23^,^24^,Orkun et al., 2022,[19]. Of the thirteen species of *Haemaphysalis* ticks reported from Pakistan [24], *Hae. sulcata* is known to transmit Spotted Fever Group (SFG) *rickettsia*. Additionally, *Hae. punctata* and *Hae. Sulcata* have both been reported to transmit piroplasmosis to cattle, buffalo, sheep, and goats in Pakistan [25,26].

*Colpodella* spp., a free-living microorganism closely related to apicomplexan parasites, represents an evolutionary bridge between free-living protozoa and parasitic apicomplexans [27,28,18]. More recently, *Colpodella* spp*.* have been reported to infect vertebrates (e.g., Amur tiger, horse) and humans,which are thought to possibly be transmitted by infected *Rhipicephalus microplus*, *Dermacentor everestianus*, and *D. nuttalli* ticks [29,30,31]. Although little is currently known about the pathogenicity, vectoral capacity, and geographical distribution of Colpodella spp., they may pose an under-recognized public health risk [32,33].

Gram-negative *Providencia rettgeri* is an opportunistic pathogen that infects immunocompromised hosts and is associated with nosocomial urinary tract infections, traveler’s diarrhea, and severe illnesses. [34,35,36,37,38]. While *P. rettgeri* has been reported to infect humans and *Drosophila melanogaster,* [39], to our knowledge, no detection in ticks has been reported. To address important gaps in the epidemiology of ticks and tick-borne pathogens in northern Pakistan, this study aimed to morphologically and molecularly characterize ticks and their associated pathogens.

## Materials and methods

### Ethics statement

The study was approved by the ethical committee of the Faculty of Life and Chemical Science, Abdul Wali Khan University Mardan.

### Study area

**Fig 1:**
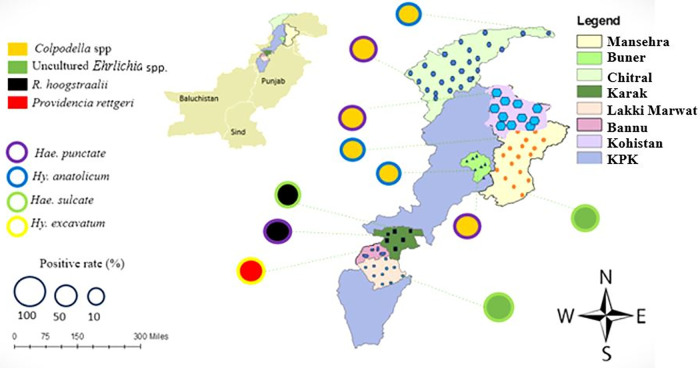
A map showing the distribution of tick collection sites throughout seven districts in Pakistan's Khyber Pakhtunkhwa province. Fig 1 was created in ArcGIS, and the base map shapefile was obtained from DIVA-GIS (https://diva-gis.org/download.html), which is openly available for use.

The study was carried out across seven districts in Khyber Pakhtunkhwa: Kohistan, Chitral, Bannu, Mansehra area, Buner, Karak, and Lakki Marwat. GPS data from these collection sites were gathered and used to create a distribution map of ticks using ArcGIS v. 10.3.1 (ESRI, Redlands, CA, USA), as illustrated in Fig 1.

### Tick collection and identification

Ticks were collected from July 2023 to June 2024, with all parts of the goats’ bodies examined for infestation. A total of 253 goats were searched, yielding 1,305 ticks. Most ticks were found in the inguinal, udder, inner surface of the thighs, and perianal and vulvar areas. After collection, distilled water was used to clean the tick specimens and were preserved in Eppendorf tubes containing 70% ethanol. These preserved specimens were then studied under a stereo-zoom microscope for morphological identification, using standard identification keys [40].

### DNA extraction and PCR

Three ticks from each host, specifically one male, one female, and one nymph were used to extract DNA. Ticks were then cleaned by using distilled water and PBS. After cleaning, specimens were allowed to dry by incubating them 25–35 minutes at 37 °C. Each tick was first cut into small pieces using sterile surgical blades and then homogenized using mortar and pestle. Genomic DNA was extracted from the homogenized tick specimens using the conventional phenol-chloroform process according to the protocol of [41]. A NanoDrop spectrophotometer (Nano-Q, Optizen, Daejeon, South Korea) was used to determine the amount of extracted DNA.

Extracted DNA was amplified by PCR utilizing the 12S rDNA partial fragment for molecular identification of ticks and the gltA, ompA, and 16S rDNA, 18S rDNA markers for pathogens associated with ticks, as indicated in Table 1. The 25 µL PCR reaction mixture was prepared using 12.5 µL of Master mix (2×) (Thermo Fisher Scientific, Inc., Waltham, MA, USA), 8.5 µL of PCR water, 2 µL of genomic DNA template (100 ng/µL), and 1 µL of each primer (10 µM) for the forward and reverse. The positive control included *Anaplasma capra* DNA (already amplified in other studies conducted in our lab), *R. massilliae* gltA DNA, and *Rh. microplus* 12S DNA. The negative control was nuclease-free PCR water. After being run on a 2% agarose gel, the amplified PCR products were visualized using the GelDoc system (BioDoc-It Imaging Systems; Upland, CA, USA) and stained with ethidium bromide. Prior to sequencing, the amplicons were purified in both directions using the Invitrogen JetFlex DNA purification kit (Waltham, MA, USFompbA).

**Table 1:** Primer’s sequences and amplicon sizes used in PCR amplification of ticks and their associated pathogens species.

Target organisms (Genetic markers)	Sequences (5′ to 3′)	Amplicon sizes (bp)	References
Tick (12S rDNA)	5’-GAGGAATTTGCTCTGTAATGG -3’	337 – 335	[62]
	5’-AAGAGTGACGGGCGATATGT-3’		
*gltA*	5’-GGGGACCTGCTCACGGCGG-3′	381	Qi et al. [42]
	5’-ATTGCAAAAAGTACCGTAAACA-3′		
ompA	5’- -3’ 5’-GCGAAATCCAAGGTACAGG-3′ 5’-ACTATTAAAGGCTAGGCTA-3′	379	[63]
EHR16S rDNA	5’-GGTACC(C/T) ACAGAAGAAGTCC-3′	345	[64]
	5’-TAGCACTCATCGTTTACAGC-3′		
BTH 18S 1st F BTH 18S 1st R	5’-GTGAAACTGCGAATGGCTCATTAC-3’ 5’-GGCTCATTACAACAGTTATAGTTTATTTG-3’	1400-1600	[Masatani et al., 2017]
BTH 18S 2nd F BTH 18S 2nd R	5’-AAGTGATAAGGTTCACAAAACTTCCC-3’ 5’-CGGTCCGAATAATTCACCGGAT-3’		

### Sequencing and phylogenetic analysis

ABI Prism 310 Genetic Analyzer capillary sequencer (Applied Biosystems) was utilized to do a bidirectional sequencing of all positive PCR products of the expected size. FinchTV (version 1.4) was used to trim the acquired sequences in order to remove primer contamination and low sequencing reads. A single consensus sequence was generated from the forward and reverse sequences of each sample. Using GenBank as a source for comparison, similar sequences [42] with higher identity were found using the Basic Local Alignment Search Tool (BLASTn; National Centre for Biotechnology Information [NCBI]). The sequences were first trimmed using FinchTV (version 1.4.0) to remove primer-contaminated areas and any misread nucleotides at the beginning and end of sequences and aligned using the ClustalW algorithm in MEGA11 (Molecular Evolutionary Genetics Analysis). The maximum likelihood technique was employed to create separate phylogenetic trees for tick and pathogen sequences, with nodes subjected to a 1000-replicate bootstrap resampling process for improved accuracy.

### Statistical analysis

All data related to tick infestation were inserted into MS Excel spreadsheet (version 2108). The data were then analyzed in Excel to calculate the total prevalence: (infested goat/total goat) × 100; the overall mean intensity: total ticks / infested goat; and the mean abundance: total ticks / total goat.

## Results

### Tick collection and infestation prevalence

A total of 253 goats (*Capra hircus*) were examined for tick infestation across various districts of Khyber Pakhtunkhwa, Pakistan. Ticks were collected from 170 goats, yielding a total of 1,305 ticks. This corresponds to an overall infestation prevalence of 67.2%. The mean number of ticks per infested goat was 7.7, and the overall mean abundance was calculated as 5.2 ticks per goat. Tick infestations were recorded year-round, with peak activity noted between May and October. District level data indicated that Buner had the highest number of ticks, followed by Kohistan, Chitral, Mansehra, Bannu, Karak, and Lakki Marwat (Fig 1).

**Fig 2:**
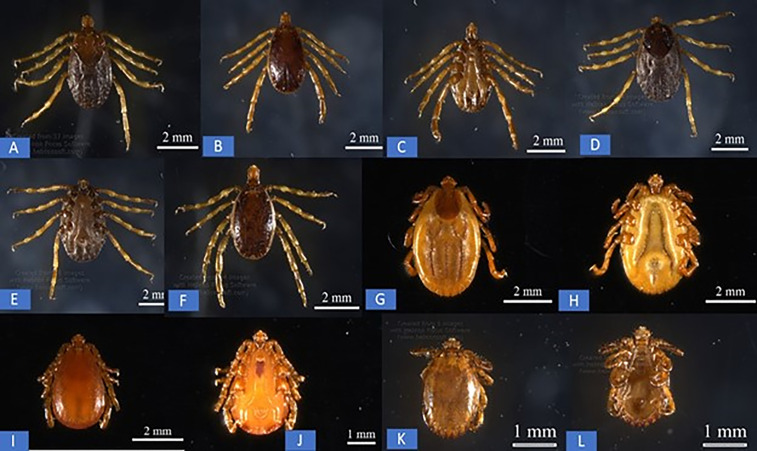
Microscopic examination of the external morphological of ticks. *Hyalomma anatolicum* female **(A)**, *Hyalomma anatolicum* male **(B)**, *Hyalomma anatolicum* male ventral side **(C)** *Hyalomma excavatum* female **(D)**, *Hyalomma excavatum* female ventral side **(E)**, *Hyalomma excavatum* male **(F)**, *Haemaphysalis sulcata* female **(G)**, *Haemaphysalis sulcata* female ventral side **(H)**. *Haemaphysalis sulcata* male **(I)**, *Haemaphysalis sulcata* male ventral side **(j)**, *Haemphysalis punctata* male **(K)**, *Haemphysalis punctata* male ventral side **(L)**, Dorsal and ventral view of ticks collected from goats showing key morphological features.

The developmental stages of the ticks included 470 adult females, 375 adult males, 281 nymphs, and 179 larvae. The detailed distribution of tick stages by district is presented in Table 2. All collected ticks were morphologically identified as *Hae. sulcate*, *Hae. punctata, Hy. anatolicum,* and *Hy. excavatum* (Fig 2) by using an established key [40].

**Table 2:** Overall tick distribution throughout Khyber Pakhtunkhwa districts, as well as tick sex and developmental phases.

Districts	Female	Male	Nymph	Larvae	Total
Buner	107	90	66	54	317
Kohistan	97	68	56	43	264
Chitral	77	57	41	29	204
Mansehra	61	46	44	25	176
Bannu	55	51	26	5	137
Karak	39	34	29	12	114
Lakki Marwat	34	29	19	11	93
**Total**	470	375	281	179	1305

### Molecular screening and overall infection rate

Of the 1,305 collected ticks, 510 were selected for molecular screening using PCR. This subset consisted of 170 females, 170 males, and 170 nymphs. Out of these, 74 ticks tested positive for at least one pathogen, resulting in an overall infection rate of 5.67%. The screening revealed the presence of four main pathogens: *Colpodella* spp., uncultured *Ehrlichia* spp., *R. hoogstraalii*, and *P. rettgeri*. Female ticks exhibited a higher infection rate than males and nymphs, with 38 positive females compared to 26 males and 10 nymphs. This trend was consistent across most districts, suggesting a sex-related difference in pathogen carriage.

### Pathogen detection

Molecular screening revealed the presence of multiple tick-borne pathogens, with *Colpodella* spp. being the most frequently detected. This protozoan was primarily found in *Hy. excavatum* collected from Buner and Kohistan, with prevalence rates of 8.98% (15/167) and 5.48% (9/164), respectively (Table 3). In Chitral, *Colpodella* spp. was also detected in *Hy. anatolicum*, where 8 of 100 ticks tested positive (8%). Uncultured *Ehrlichia* spp. was detected exclusively in *Hae. sulcata*, with 12 out of 176 ticks (6.81%) in Mansehra and 10 out of 93 ticks (10.75%) in Lakki Marwat testing positive, indicating the pathogen’s circulation in both regions. The highest pathogen prevalence observed in the study was in Karak, where *Hae. punctata* carried *R. hoogstraalii* at a rate of 14.28% (12/84), marking the district as a hotspot for rickettsial infections. In Bannu, *Hy. excavatum* was found to harbor *P. rettgeri*, with a prevalence of 5.83% (8/137), suggesting the emergence of this bacterium as a potential zoonotic agent.

**Table 3:** Information regarding tick species, number of ticks collected, locality, and the molecular detection of pathogens in these tick species.

District	Tick species	Count	Ticks subjected to PCR (F, M, N,)	Screened pathogens	Pathogens detection in tick species
Buner	*Hy. excavatum*	167	72 (24, 24,24)	8F; 5M; 2N	15/167 (8.98%). *Colpodella* spp.
	Hae. *punctata*	150	60 (20, 20, 20)	–	–
Kohistan	*Hy. excavatum*	164	51 (17, 17, 17)	5F; 3M; 1N.	9/164(5.48%). *Colpodella* spp.
	Hae. *punctata*	100	48 (16, 16, 16)	–	–
Chitral	Hae. *punctata*	104	42 (14, 14, 14)	–	–
	*Hy. anatolicum*	100	42 (14, 14, 14)	4F; 4M	8/100 (8%). *Colpodella* spp.
Mansehra	Hae. *sulcate*	176	72 (24, 24,24)	5F; 4M; 3N	12/176 (6.81%). Uncultured *Ehrlichia* spp.
Karak	Hae. *punctata;*	84;	36 (12, 12, 12)	6F; 4M; 2N	12/84 (14.28%) *R. hoogstraalii*
	*Hy. anatolicum*	30	12 (4, 4, 4)	–	–
Lakki Marwat	Hae. *sulcata*	93	42 (14, 14, 14)	5F; 3M; 2N	10/93 (10.75%) Uncultured *Ehrlichia* spp.
Bannu	*Hy. excavatum*	137	33 (11, 11, 11)	5F; 3M	8/137 (5.83%). *Providencia rettgeri*
Total		1305	510 (170, 170, 170)	38F; 26M; 10N	74/1305 (5.67%)

Host-pathogen-tick associations summarized in Table 4 show that *Hy. anatolicum* from Buner, Chitral, and Kohistan was associated with *Colpodella* spp., *Hae. punctata* from Karak with *R. hoogstraalii*, *Hae. sulcata* from Mansehra and Lakki Marwat with *Ehrlichia* spp., and *Hy. excavatum* from Bannu with *P. rettgeri*. Overall, 74 out of 510 ticks (5.67%) tested positive, highlighting the complex and region-specific distribution of tick-borne pathogens and underscoring the importance of ongoing molecular surveillance to understand and mitigate potential zoonotic risks in livestock-rearing regions of Pakistan.

**Table 4:** Shows pathogen positive ticks and its associated pathogens recorded from different locations and host across the study region.

Host	Region	Tick	Tick Accession Number	Pathogens and their GenBank Accession no			
				*Colpodella* spp.	*P. rettgeri*	*R. hoogstraalii*	Uncultured *Ehrlichia* spp.
Goat	Buner	*Hy. anatolicum*	OR665366	OR672117 PV412596	–	–	–
	Chitral	*Hy. anatolicum*	OR665367	OR672113 PV412595	–	–	–
	Kohistan	*Hy. anatolicum*	OR665373	OR672115	–	–	–
	Bannu	*Hy. excavatum*	OR665368	–	OR899980	–	–
	Karak	*Hae. punctata*	OR665360	–		OR899979, OR899976	–
	Lakki Marwat	*Hae. sulcata*	OR665361, OR665362, OR665363	–	–	–	OR668793
	Mansehra	*Hae. sulcata*	OR665364, OR665365	–	–	–	OR668794

### Ticks and pathogens DNA analysis

*Haemaphysalis sulcata*, *Hae. punctata, Hy. anatolicum* and *Hy. excavatum* ticks were used to extract DNA. All tick-related sequences, together with their pathogen, were uploaded to the NCBI's GenBank (Table 4). The *Hae. sulcata* 12S rDNA amplicons showed a 99–100% similarity range for 12S rDNA in the BLAST analysis's, with a percent identity of 99.15% with the *Hae. sulcata* partial 12S rDNA sequence from Algeria (KY511421), *Hae punctata* display 100% identity with *Hae. punctata* sequence from China (MN267437), *Hy anatolicum* display 100% identity with *Hy. anatolicum* sequence from Pakistan (OR911528) and *Hy. excavatum* displays 100% identity with *Hy. excavatum* reported from Turkey (MG418642).

BLAST analysis revelated the 16S rDNA sequence of the *Colpodella* spp*.* species isolated from *Hy. anatolicum* displayed a higher percent identity to other *Colpodella* spp*.* species reported in GenBank. Specifically, the species was found to have 100% identity and query cover with sequence *Colpodella* spp. (MH208621) isolated from *Rhipicephalus haemaphysaloides* in China, and 99.92% with *Colpodella* spp. (GQ411073.1) that isolated from woman in China with relapsing Babesia-like illness. Similarly, the sequence had a 99.59% identity to *Colpodella* spp. (MH012046.1) that isolated from *Dermacentor nuttalli* in China. The gltA amplified *P. rettgeri* yielded 99.04% to *Providencia* spp. from the urine of human in China, and 99.03% (CP076406) with a patient isolate from Argentina. The gltA amplified *R. hoogstraalii* showed 100% similarity (MF383601) with an isolate from *Hae parva* removed from a patient in Turkey, and 98.33% (KY570489) with an isolated from ticks in Greece, and uncultured *rickettsia* (MT502507) from South Korea. The Uncultured *Ehrlichia* spp. 16S (OR668794) shows 100% identity with (OP047595) Uncultured *Ehrlichia* spp. that was isolated from human in USA. Details on pathogens detected by district can be found in Table 4.

### Phylogenetic analysis of tick sequences

**Fig 3:**
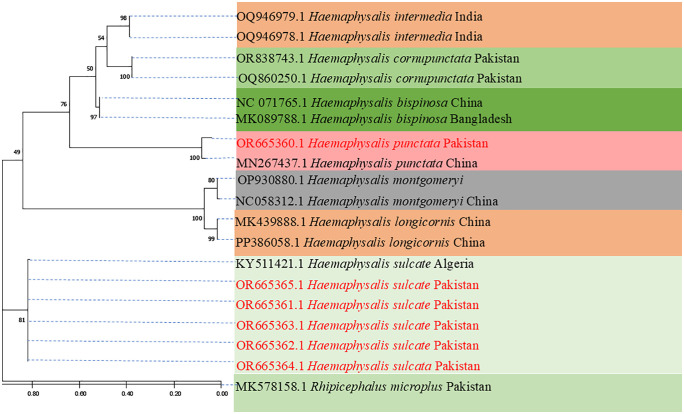
Phylogenetic tree of *Hae. sulcata* and *Hae. punctata* based on 1000 bootstrap iterations of the incomplete 12S sequence using the Maximum Likelihood method and the General Time Reversible (GTR + G + I) model.

**Fig 4:**
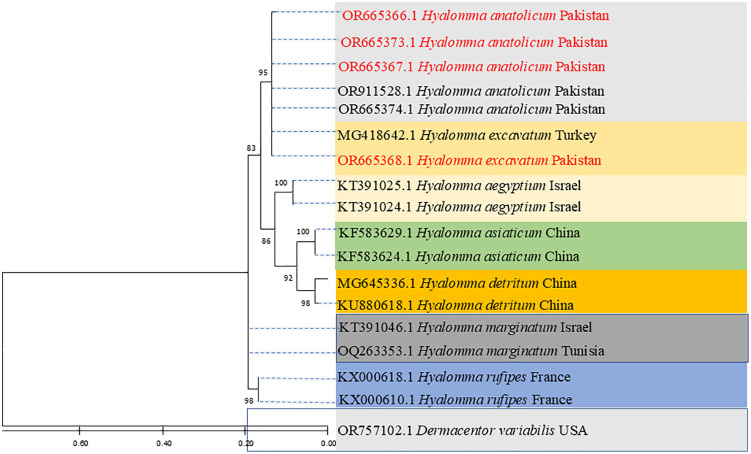
The Maximum Likelihood technique, using 1000 bootstrap iterations of the incomplete 12S sequence, was used to create the phylogenetic tree of *Hy. anatolicum* and *Hy. excavatum.*

In the current investigation, the haplotype grouped with the 12S sequence of *Hae. sulcata* that had previously been reported from Algeria, according to the evolutionary tree that was created by using 12S partial ribosomal DNA (KY511421). All 5 sequences of *Hae. sulcata* reported from this study clustered together with same number of nucleotide substitutions in the same clade. The *Hae. punctata* in this study grouped with the *Hae. punctata* 12S sequence that was previously published from China (MN247437). A number of *Haemaphysalis* spp. sequences were included in the phylogenetic tree as references. Additionally, *Rh. microplus* 12S sequence reported from buffaloes of Pakistan (MK578158) were used as an outgroup in the current tree as shown in Fig 3. The evolutionary tree inferred for the partial 12S partial ribosomal DNA of *Hy. anatolicum* in the current study revealed the study’s haplotype grouped with the *Hy. anatolicum* 12S sequence that had been reported from Pakistani goats (OR911528, OR665374). All 3 sequences of *Hy. anatolicum* reported from this study clustered together with same number of nucleotide substitutions in the same clade. The *Hy. excavatum* of the current study clustered together with the 12S sequence of *Hy. excavatum* previously reported from Turkey (MG418642). The other *Hyalomma* sequences were included in the phylogenetic tree as references. Similarly, the 12S sequence of *D. variabilis* reported from USA (OR665368) were used as an outgroup in the inferred tree as shown in Fig 4.

### Phylogenetic analysis of pathogen sequences

**Fig 5:**
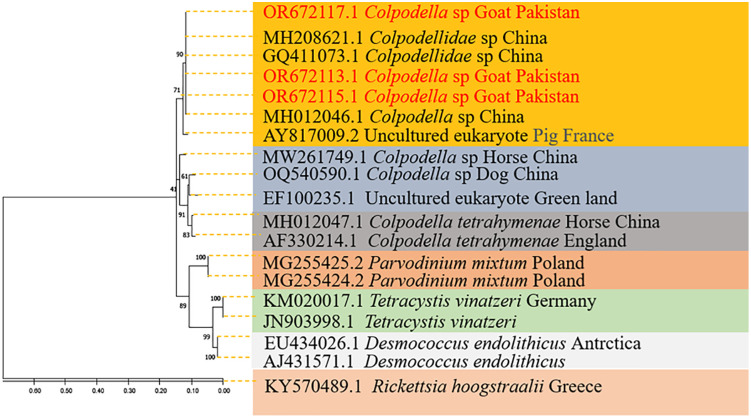
The phylogenetic tree for *Colpodella* spp. was inferred using the Maximum Likelihood technique. Pathogens amplified using the 16S rDNA partial fragment sequence, 1000 bootstrap iterations, and the Kimura 2 parameter with Gamma distributions (+G).

The Maximum Likelihood inferred phylogenetic tree of *Colpodella* spp. produced several clades of the genus *Colpodella* spp. The current study’s haplotype grouped with similar species reported from *Rh. haemaphysaloides* in China (MH208621) and *Colpodella* spp. from woman with relapsing Babesia-like illness (GQ411073) in China. The *Colpodella* spp. sequence also clustered with *Colpodella* spp*.* that was detected in *D. nuttalli* in China (MH012046) as well as with an uncultured *eukaryote* from France (AY817009) as shown in Fig 5. The tree uses several other species in the same genus for referencing the current haplotype. All the *Colpodella* spp. were clustered with 99.8% ultra-fast bootstraps support values and a 97% approximate likelihood ratio iterated 1000 times. The *R. hoogstraalii* species reported from a tick in Greece (KY570489) used as an outgroup as shown in Fig 5.

**Fig 6:**
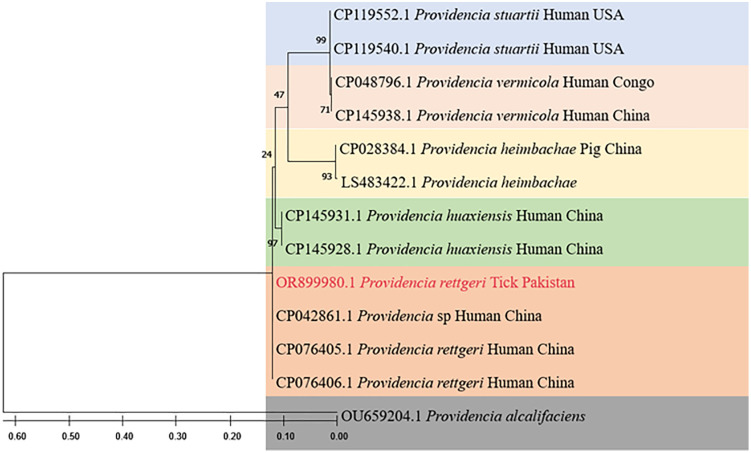
The partial CDS sequence of the citrate synthase (gltA) gene served as the basis for the construction of the phylogenetic tree of *P. rettgeri*, which was confirmed over 1000 bootstrap iterations using the Maximum Likelihood approach with the Tamura 3-parameter model and Gamma distribution (+G).

The *P. rettgeri* phylogenetic tree was inferred using maximum likelihood method. The tree resulted in several clades of the *P. rettgeri* species. The current haplotype grouped with the same species *P. rettgeri that* reported from human in China (CP076405), (CP076406). The sequences also clustered with *Providenica* previously reported from human in China (CP042861). These clustered species had different nucleotide substitutions which likely presents the distinct genotype nature of the reported species shown in Fig 6. *Providenica alcalifaciens* species (OU659204) were used as an outgroup for referencing.

**Fig 7:**
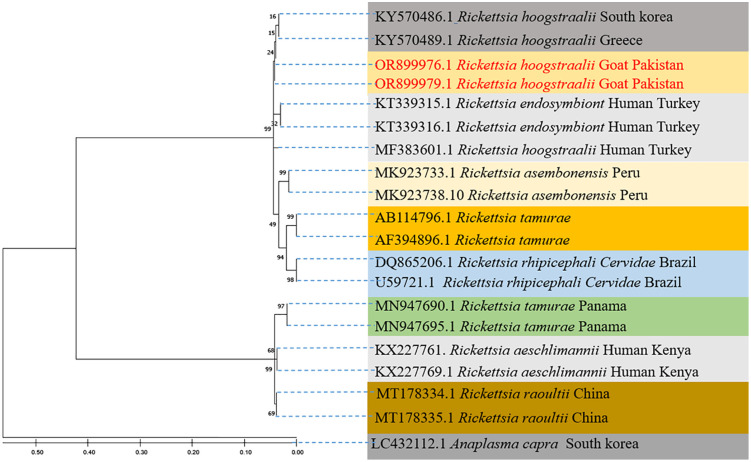
The phylogenetic tree of *R. hoogstraalii* was generated using the Maximum Likelihood technique with the Tamura 3-parameter and Gamma distribution (+G) and 1000 bootstrap iterations based on the partial CDS sequence of the citrate synthase (gltA) gene.

The highest likelihood approach was used to infer the evolutionary tree of *R. hoogstraalii*. The tree resulted in several clades of the *R. hoogstraalii* species. The current haplotype grouped with the same species *R. hoogstraalii* that reported from *Hae. parva* ticks (KY570489) and a patient (KY570486) in Greece, as shown in Fig 7, with *A. capara* from South Korea (LC432112) used as an outgroup for referencing.

**Fig 8:**
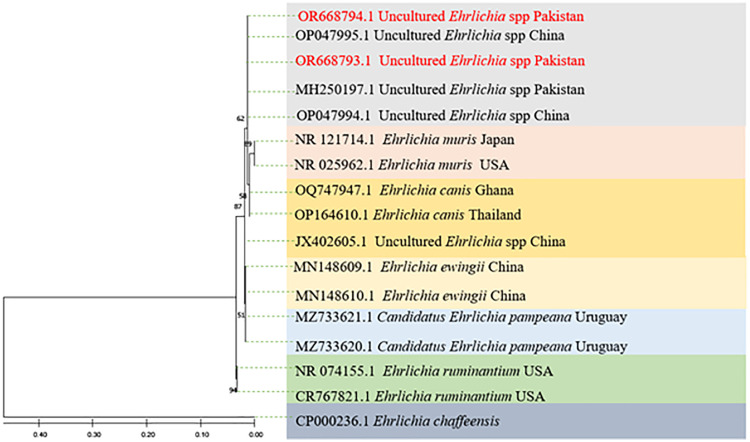
Using a partial fragment sequence of the 16S rDNA, 1000 bootstrap rounds of pathogens amplified, the phylogenetic tree of uncultured *Ehrlichia* spp. was constructed using the Maximum Likelihood technique with the Kimura 2 parameter and Gamma distributions (+G).

The phylogenetic tree for uncultured *Ehrlichia* spp. was inferred using maximum likelihood method. The tree resulted in several clades of the uncultured *Ehrlichia* spp. The current haplotype grouped with the same species of uncultured *Ehrlichia* spp*.* that reported from *Rh. microplus* in China (OP047995), (OP047994). The sequences were also clustered with uncultured *Ehrlichia* spp. that previously reported from *Hy. anatolicum* in Pakistan (MH250197) as shown in Fig 8, using *E. chaffeensis* (CP000236) as an outgroup.

**Fig 9:**
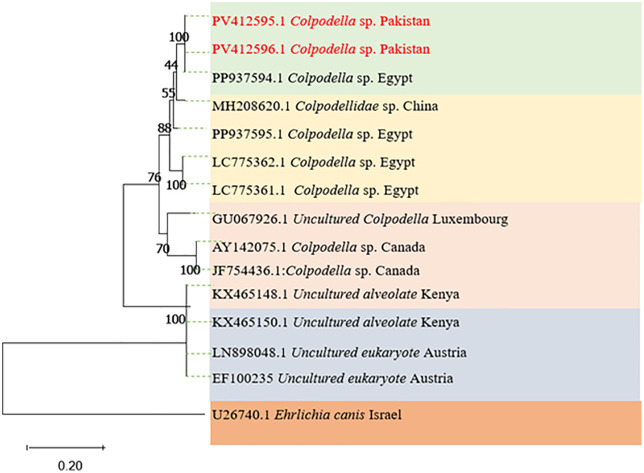
The phylogenetic tree for *Colpodella* sp. was inferred using the Maximum Likelihood technique. Pathogens amplified using the 18S rDNA partial fragment sequence, 1000 bootstrap iterations, and the Kimura 2 parameter with Gamma distributions (+G).

The Maximum Likelihood inferred phylogenetic tree of *Colpodella* spp. produced several clades of the genus *Colpodella* spp. The current study’s haplotype grouped with similar species reported from *Rh. annulatus* in Egypt (PP937594) and *Colpodella* spp. from (MH208620) in China. The *Colpodella* spp. sequence also clustered with *Colpodella* spp*.* as shown in Fig 9. The tree uses several other species in the same genus for referencing the current haplotype. All the *Colpodella* spp*.* were clustered with 99.8% ultra-fast bootstraps support values and a 97% approximate likelihood ratio iterated 1000 times. The *Ehrlichia canis* reported from tick in Israel (U26740) were used as an outgroup for referencing.

## Discussion

This study highlights the significant burden of tick infestations and the associated risk of tick-borne pathogens (TBPs) in Pakistan, with implications for public health and livestock productivity. A high tick infestation prevalence (67.19%) was recorded in goats, with the Buner district showing the highest proportional infestation rate (77.19%). Morphological and molecular analyses identified four tick species (*Hy. anatolicum, Hy. excavatum, Hae. sulcata,* and *Hae. punctata*). Pathogen analysis revealed the presence of zoonotic agents such as *R. hoogstraalii* and novel or emerging pathogens like *Colpodella* spp*.* and *P. rettgeri,* marking the first potential detection of *Colpodella* spp*.* in Pakistan. Seasonal activity peaking from May to October further accentuates the risk of zoonotic disease transmission during these months.

The current study provides insights into the distinct pathogen profile of the various tick species infesting goats of Pakistan, including the detection of opportunistic pathogenic bacteria, *P. rettgeri* [43,44] was isolated from *Hy. excavatum*. Additionally, *Colpodella* spp*., R. hoogstraalii* and uncultured *Ehrlichia* spp., were also detected in *Hy. anatolicum*; Hae. *sulcata*, and *Hae. punctata* ticks.

*Colpodella-*like species have been recently reported as potential infectious agents and causes human infections [45,46], with the first human infection caused by *Colpodella* spp., through tick bites being reported in China in 2012 [47,48]. This emerging species, *Colpodella* spp., has previously been reported in *Ixodes persulcatus, Rh. microplus, Rh. haemaphysaloides, Hae. longicornis*, and *Hy. dromedarii* [31,32,49,50]. The findings of this study make a case for expanded surveillance of this microorganism in *Hy. anatolicum* ticks as a possible carrier of *Colpodella* spp*.* Additionally, monitoring atypical neurological symptoms following tick bites for possible *Colpodella* infection will help determine if this pathogen poses a risk to human populations in Pakistan.

Although *R. hoogstraalii* belongs to the SFG Rickettsia group, little information about its pathogenicity in vertebrates is currently available [51]. The first instance of isolation of *R. hoogstraalii* happened in 2006, from *Hae. sulcata* ticks infesting goats and lambs in Croatia. Hard ticks from both domestic and wild ruminants in Europe have also been shown to contain *R. hoogstraalii*. Ticks from various locations in Europe have been found to carry *Rickettsia hoogstraalii*, including *Hae. punctata* and *Hae. sulcata* from Sardinia, Italy and Spain; *Hae. parva* and *Hae. sulcata* from Greece, *Hae. sulcata* and *Dermacentor marginatus* from Georgia, and *Hae. punctata* from Cyprus [23,52,53,54]. It has also been found in soft tick species, in Ethiopia, Japan, Iran, Namibia, Zambia, China, and the United Arab Emirates [55,56,57,58,59,60,61]. Previous research has revealed that multiple species of Rickettsia have been found in Pakistan in ticks that infest a variety of hosts. However, very limited information about the presence and genetic characterization of *R. hoogstraalii* has been reported. Here we describe the first report of *R. hoogstraalii* detected in Hae. *punctata* in Pakistan*.* Further research should be done to assess the pathogenicity of *R. hoogstraalii* in mammals as well as the potential role of *Hae. punctata* as a competent vector of disease.

### Limitations

This study provides the first molecular evidence of *Colpodella* spp*.*, *R. hoogstraalii*, and *P. rettgeri*, in ticks infesting goats in Pakistan; however, several limitations must be acknowledged. First, due to the engorged status of some of the collected ticks, there is a possibility that the detected pathogens particularly *P. rettgeri* originated from the goat host's blood meal rather than representing true tick infections. This raises the concern of environmental or host-derived contamination, especially in the case of *P. rettgeri*, which is known to be an opportunistic pathogen in diverse environments. Second, the study utilized relatively short gene fragments (gltA and 16S rRNA) for pathogen detection and identification. While these markers are commonly used for preliminary screening, sequencing longer genomic regions or using whole-genome approaches in future studies would provide more robust taxonomic resolution and support for pathogen identification. We also acknowledge the need to determine what role, if any, ticks play in transmission of *Coplodella* spp. and *P. rettgeri* in Pakistan, which should include pathogen localization within tick tissues and experimental transmission studies.

## Conclusions

In conclusion, this study adds to our understanding of pathogen diversity within common tick species infesting goats in Pakistan. The identification of *P. rettgeri*, *Colpodella* spp*.*, and *R. hoogstraalii* underscores the importance of recognizing emerging and potentially zoonotic pathogens in local tick populations. The detection of *Colpodella* spp*.* in *Hy. anatolicum* ticks highlights the need for further investigation into the vector competence of ticks and the possible zoonotic transmission of this pathogen. Additionally, the first report of *R. hoogstraalii* in *Hae. punctata* from Pakistan represents a new data regarding its geographic distribution and potential tick vector. These findings underscore the urgency of conducting further research on the pathogenicity, transmission dynamics, and ecological factors influencing tick-borne diseases in Pakistan. The results of this study provide a critical foundation for future investigations into tick-borne pathogens and emphasize the necessity of continuous surveillance and public health efforts to mitigate the risks associated with emerging infectious diseases.
